# Significant Shear Failure Difference among Additively Manufactured Polymers Using Different Techniques

**DOI:** 10.3390/polym14194028

**Published:** 2022-09-26

**Authors:** Luoyu Roy Xu, Qinglin Wang, Yinxu Ni, Gonghe Zhang, Fenghua Liu, Xiaodong Zheng, Yang Liu

**Affiliations:** 1 School of Mechanical Engineering and Mechanics, Ningbo University, Ningbo 315211, China; 2 MOE Key Laboratory of Impact and Safety Engineering, Ningbo University, Ningbo 315211, China; 3 Zhejiang Key Laboratory of Additive Manufacturing Materials, Ningbo Institute of Materials Technology and Engineering, Chinese Academy of Sciences, Ningbo 315211, China

**Keywords:** shear strength, polymer, fused deposition modeling, selective laser sintering

## Abstract

Because additively manufactured materials are increasingly being used in load-bearing structures, strength research has become critical. Surprisingly, numerous studies have reported the tensile strength measurements, but only a few studies have presented meaningful results for the shear strength measurements of additively manufactured polymers. Hence, this paper proposes a combined experimental and numerical investigation of a new interlayer shear strength measurement approach, and it targeted the applications of the same polyamide (PA12) specimens made with fused deposition modeling (FDM) and selective laser sintering (SLS). A necking-shaped shear specimen was developed to measure the pure shear strengths with the aid of a three-dimensional (3D) finite element analysis. The results showed that the specimens made with FDM and SLS exhibited totally different shear failure behaviors. The ultimate shear strength of the FDM-PA specimens had more than a 32% increase over that of the SLS-PA specimens. An interface mechanics assumption was employed to explore the different shear failure mechanisms with the support of a fractography analysis.

## 1. Introduction

Additive manufacturing (AM)/3D printing technologies have become a key focus of recent materials and manufacturing research. Because some additively manufactured materials are used in load-bearing structures, their strength and failure research is critical [1,2,3,4]. Although the tensile strength is among the most important mechanical properties of engineering materials, the shear strength is also an important material property, particularly because it is not easy to measure. Generally, a structure has a combined stress state, i.e., the normal and shear stresses coexist. Therefore, both the tensile and shear strengths must be accurately determined before the extensive application of any additively manufactured material. Surprisingly, numerous studies have reported the tensile strength measurements for additively manufactured materials, but few studies have presented meaningful shear strength measurements. Hence, a new approach for measuring the potential low interlayer shear strength is worth exploring.

There are many kinds of AM technologies for different materials. However, a basic question is often asked: are mechanical behaviors the same or different for the same-material specimens made with different AM technologies? This pilot study was intended to explore the above issue using one additively manufactured polymer material. The study is novel because no previous papers addressed this issue. Moreover, this topic becomes important when many AM techniques are developed and we have to identify which AM technique can improve (or damage) the mechanical properties of printing materials. 

## 2. Materials and Methods

### 2.1. New Necking-Shaped Shear Test

**Figure 1:**
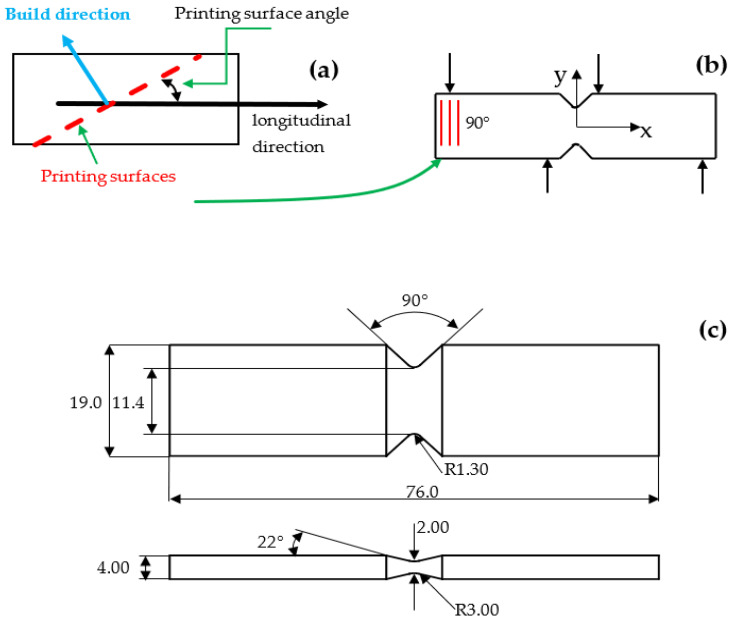
(**a**) Definition of the printing surface angle. (**b**) Shear specimen with a printing surface angle of 90°. (**c**) Necking-shaped shear specimen with a variable central cross-section (all dimensions in mm).

This research was focused on the interlayer shear strength measurement of the same polyamide (PA12), which was fabricated with FDM and SLS. As shown in Figure 1, a specimen with a printing surface angle of 90° was employed. The printing surfaces refer to the interfaces between two printing layers. In fact, 3D printing materials have an intrinsic build direction (perpendicular to the printing surfaces). However, a reference system is needed to specify an exact angle. In this study, the shear specimen’s longitudinal/horizontal direction was chosen as the reference. For the mechanics analysis, the printing surface direction was more convenient than the build direction. Because the shear force acted along the printing surface, the measured shear strength was the interlayer shear strength.

Inspired by the Iosipescu shear (IS) test (ASTM standard D5379-2019) that demonstrated a nearly constant shear stress variation at the specimen center with a rounded V-notch [5,6], we proposed a necking-shaped shear specimen with a variable central cross-section area (Figure 1c). The term “necking” was chosen because plastic deformation occurred before the final shear failure. Because of the small central cross-sectional area, the necking-shaped specimen center had a large shear stress value that quickly led to failure in shear. We recently conducted a series of combined experimental and numerical investigations to characterize the tensile and shear strengths of 3D printing materials [7,8]. The result reported in that paper was an unexpected and unreported outcome that was not seen in the open literature. 

### 2.2. Specimen Manufacture Using FDM and SLS

For FDM, the PA12 filaments were purchased from Shenzhen eSUN Industrial Co., Ltd., Shenzhen, China. The filament diameter was 1.75 mm (standard deviation ± 0.03 mm). The FDM 3D printer was a Raise3D Pro3 (Shanghai Fuzhi Information Technology Co., Ltd., Shanghai, China). The printing parameters and other details can be found in Zhang et al. [7].

To manufacture PA specimens using SLS, a PA12 powder with a spherical shape and a mean particle size of 120 μm was used, and the apparent density was 0.48 g/cm^3^. A selective laser sintering apparatus (HT252P, Hunan Farsoon High-Technology Co., Ltd., Changsha, China) was employed to create the specimens. The apparatus was equipped with a 60 W carbon dioxide laser with a focal laser beam diameter ≤ 0.5 mm. The processing parameters and other details can be found in Wang et al. [8].

### 2.3. Pure Shear Experiment

All of the shear specimens were tested on an Instron 5966 test frame with a 10 kN load cell. The necking-shaped shear specimen was inserted into an Iosipescu shear fixture, and the shear load was applied in the form of displacement. The displacement rate of all tests was 1 mm/min, and the maximum loadings of the specimens were recorded by the test machine. The fracture surfaces of some SLS-PA specimens were characterized using a field emission scanning electron microscope (SEM) Quanta 250.

### 2.4. Three-Dimensional Finite Element Analysis (FEA)

The FEA model of the PA shear specimen was built with ABAQUS 2020 to obtain the stress distributions. Because the theoretical printing interfaces had no thickness, the FEA models of the 3D printing polymers had the exact same models for their bulk forms, i.e., we did not need to build an FEA model for a 3D printing polymer with interfaces. The major difference was that the 3D printing polymers had different failure criteria related to their interfaces and anisotropic strengths. Fine C3D8R elements were employed to analyze the stresses of the specimen center and the contact areas with the loading blocks. Displacements were applied to the right specimen edge to simulate the realistic boundary/force conditions.

## 3. Results and Discussion

### 3.1. Stress Distribution of the Necking-Shaped Shear Specimen

**Figure 2:**
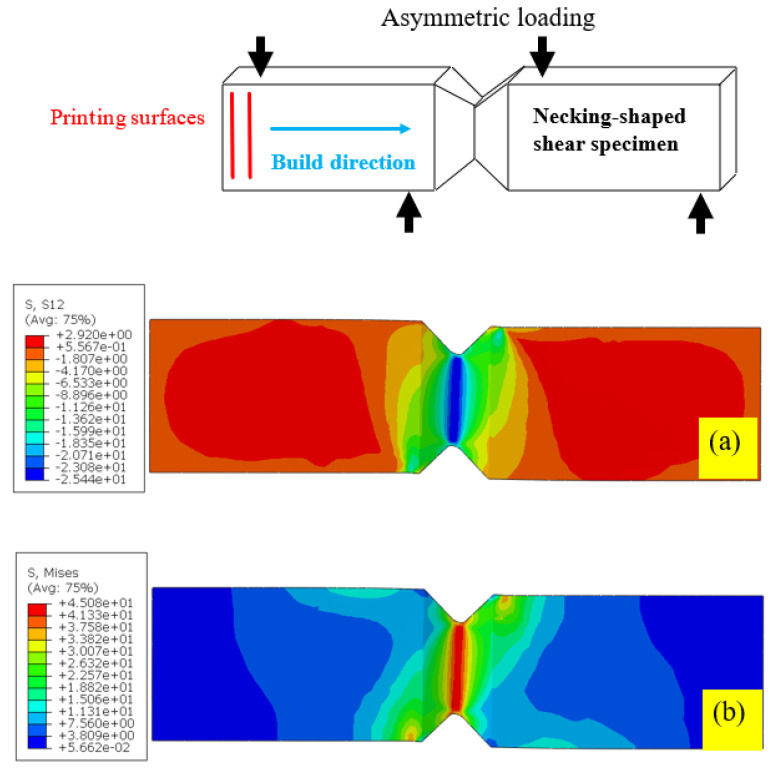
Contour plots of (**a**) shear and (**b**) von Mises stresses from the FEA of a PA necking-shaped specimen (applied displacement of 1 mm).

Figure 2 shows the contour plots of the in-plane shear stress *τ_xy_* and the von Mises stress distributions of a PA specimen when the applied displacement was 1.0 mm. Compared to the large central shear stress area and the low shear stress value of the Iosipescu shear specimen [5,8], the necking-shaped specimen had a narrow shear stress distribution at the specimen center and a high shear stress value. Therefore, this was a clear advantage of the pure shear failure test. Similar to the shear stress distribution, the von Mises stress had a narrow stress distribution at the specimen center and a high stress value, which meant that plastic deformation first occurred at the specimen center.

### 3.2. Significant Difference in Shear Failure Processes for Two Kinds of PA Specimens

**Figure 3:**
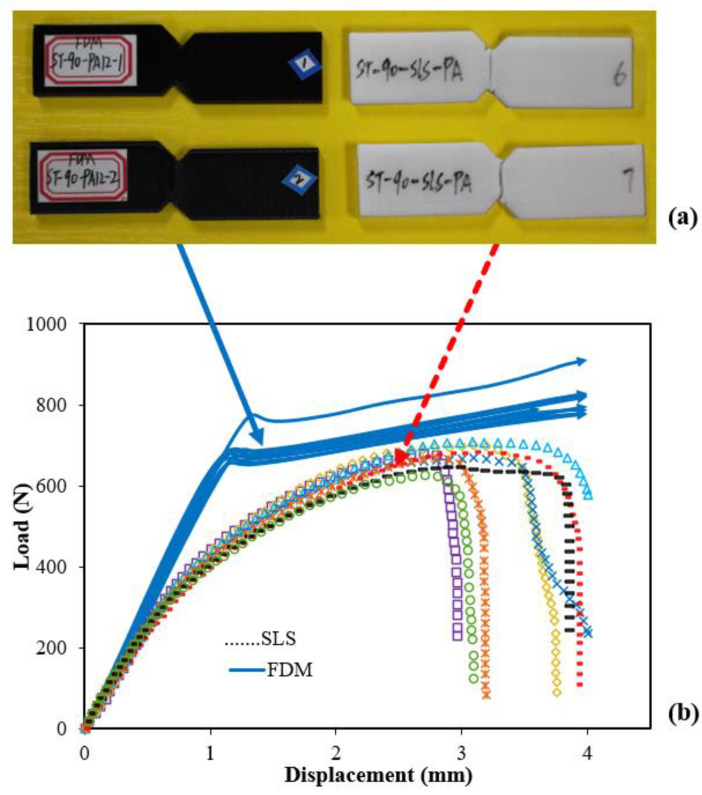
(**a**) Very different shear failure patterns and (**b**) loading processes of PA specimens made with FDM (black specimens, no fracture) and SLS (white specimens, shear fracture).

Table 1 presents the interlayer shear strength values with small standard deviations (<7%). The strengths of the same printing material were very different for the FDM-PA specimens. Figure 3a shows a quite consistent shear fracture pattern for the two SLS-PA shear specimens (white, right side) that had a straight and smooth vertical fracture path similar to other shear specimens made with FDM. However, for the FDM-PA specimens (black, left side), large plastic deformation occurred rather than fracturing. Figure 3b shows the load-displacement curves of the FDM-PA and SLS-PA specimens. These curves were highly repeatable. However, these load-displacement curves showed totally different shear failure behaviors of the same PA material manufactured using different AM techniques (FDM and SLS).

For the SLS-PA specimens, the initial linear load-displacement curves were short, and nonlinear deformation occurred until completely fracturing, which was related to the plastic deformation and was a major part of these curves. In contrast, for the FDM-PA specimens, the initial linear load-displacement curves were very long, and a yielding stage (yielding strength 27.69 MPa) was clearly identified. However, these specimens still carried loads and dissipated energy until the displacement reached the maximum limit of 4.0 mm. Therefore, the lower boundary of their ultimate strength was greater than 32 MPa (or more than a 32% increase over the SLS-PA specimens). Because the specialized IS fixture had a displacement limit and the PA specimen’s Young’s modulus (then compliance) was low, all the experiments stopped if the displacement reached its limit. These repeatable tests demonstrated the significant load-bearing advantage of the FDM-PA specimens. Hence, it was necessary to obtain a preliminary understanding of the unique failure mechanisms.

### 3.3. Shear Failure Mechanism Analysis Using Interface Mechanics and SEM

**Figure 4:**
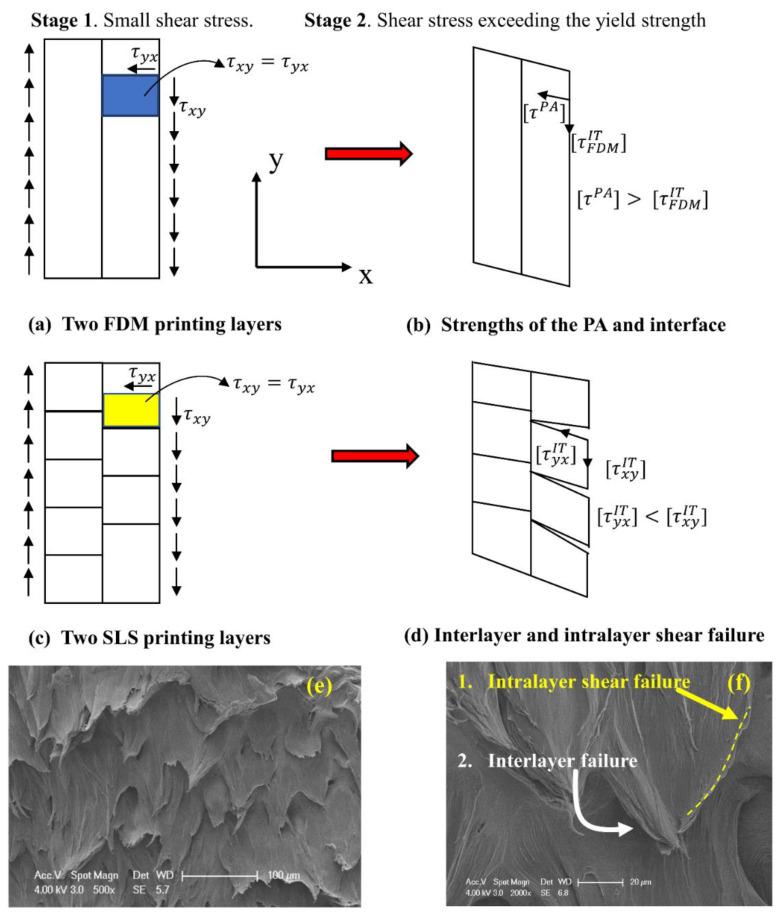
Assumed shear failure mechanisms from the side view of two printing layers of SLS and FDM specimens, (**a**) two FDM printing layers, (**b**) strengths of the original PA polymer and interface, (**c**) two SLS printing layers, (**d**) interlayer and intralayer shear failure, (**e**) SEM image of an SLS specimen, (**f**) two failure modes on an SEM image.

Figure 4 illustrates the different failure patterns of two printing layers (side view or 2D case) of the same PA material made with the two AM techniques. Two stages were assumed: (1) both specimens were subjected to a very small shear stress, and (2) the applied shear stress exceeded the yielding strength. For the FDM specimen, the printing layer was assumed to be the nozzle diameter with a layer thickness of 0.4 mm, as shown in Figure 4a. However, for the SLS specimen, the printing layer was related to the micro melt pools with a diameter of several micrometers, as shown in Figure 4c. If one representative element was taken from both specimens, it had a shear stress acting on the layer’s cross section (intralayer shear stress) $\tau_{yx}=\tau_{xy}\;\;$(shear stress acting along the interface or interlayer shear stress) according to the mechanics of the materials.

The FDM-PA specimen only had the interfaces between the printing layers and one interfacial strength $[\tau_{FDM}^{IT}$], while its shear strength related to the intralayer stress $\tau_{yx}\;\mathrm{was}\;\left[\tau^{PA}\right]$, which was the shear strength of the bulk PA, as illustrated in Figure 4b. Usually, the strength of the bulk material is larger than the strength of the same material with interfaces for layered materials [9], i.e., $\left[\tau^{PA}\right]>\left[\tau_{FDM}^{IT}\right]$. The SLS-PA specimen had weak interfaces inside the layer and between the layers formed by the melted spherical particles. Therefore, it had two interfacial strengths, and we assumed that its intralayer shear strength was $[\tau_{yx}^{IT}]<[\tau_{xy}^{IT}$] (interlayer shear strength), as illustrated in Figure 4d.

The shear strength variation had two steps: (1) strength at the yielding and (2) strength at the final failure. In step 1, the FDM-PA specimen had interfacial plastic deformation, but it still could carry a load until failure because of the strength relationship, as illustrated in Figure 4b. However, the SLS-PA specimen was completely different because we assumed that the intralayer shear failure occurred first, which then induced the interlayer shear failure, as shown in Figure 4d. Then, the shear failure elements formed along the shear force direction.

The SEM images of the SLS-PA specimens provided some preliminary evidence for our assumed failure processes. As shown in Figure 4e, microtearing surfaces with curling edges along the shear force direction were found to be homogeneously distributed on the whole surface. Further details are shown in the amplified SEM image in Figure 4f, which shows two kinds of shear failure. The right edge of the tearing piece was believed to be an intralayer shear fracture path. It is important to note that the tearing piece tip was above the surface. Therefore, this image supported our assumption that the interlayer shear failure that separated the tearing piece from the printing layer underneath occurred after the intralayer shear failure, as illustrated in Figure 4d. These failure sequences have often been observed in some layered materials with interfaces [9,10]. In contrast, if the interlayer shear failure was initiated before the intralayer shear failure, the shear fracture surface would be quite smooth. Recently, we employed a Ti-6Al-4V alloy powder to manufacture shear strength specimens using the selective laser melting technique, and we found that the shear fracture surface was very smooth and completely opposite to that shown in Figure 4e, showing many rough tearing pieces. Of course, the actual shear failure process in this study was extremely complicated; so, our purpose was to promote immediate interest from interdisciplinary researchers. Specifically, not many studies have focused on the shear failure of 3D printing polymers [11,12,13]. Our future work will be focused on the shear crack propagation of the same PA12 material, but the specimens will be made with SLS and FDM. 

The above phenomenon is extremely important for engineering applications because the FDM-PA specimens not only carried a large load but also dissipated more energy. The nature of the different shear failure processes was related to the large filaments and small powder/particles of the AM techniques. Similar to composite materials reinforced by long fibers or particles, FDM using large filaments have better strengths than SLS. Our recent tension experiments also showed that the strengths of the injection-molded PA, FDM-PA, and SLS-PA were 43.68 MPa (100%), 42.83 MPa (98.1%), and 38.48 MPa (88.1%), respectively, i.e., the specimens made with SLS always had low strengths [7,8]. Therefore, although the advantages of SLS might be obvious, its weakness should receive enough attention as well. 

## 4. Conclusions

A necking-shaped shear specimen was developed to measure the pure shear strengths with the aid of a 3D finite element analysis. The ultimate shear strength of the FDM-PA specimens had more than a 32% increase over that of the SLS-PA specimens, which can be explained by a proposed interface mechanics model with the support of the fractography analysis. Therefore, AM techniques should focus on the constituents and sizes of AM elements, and the SLS technique may significantly reduce mechanical properties.

## Figures and Tables

**Table 1:** Interlayer shear strengths of the same PA 12 polymer made with SLS and FDM.

Manufacturing Technique	Strength (MPa)	Specimen Number	Remarks
FDM	27.69 ± 1.53 (yielding)	10	Ultimate strength > 32 MPa
SLS	24.24 ± 1.17	8	Pure shear failure

## Data Availability

The data presented in this study are available on request from the corresponding author.
